# Novel therapeutic strategies targeting telomere maintenance mechanisms in high-risk neuroblastoma

**DOI:** 10.1186/s13046-020-01582-2

**Published:** 2020-05-06

**Authors:** S. L. George, V. Parmar, F. Lorenzi, L. V. Marshall, Y. Jamin, E. Poon, P. Angelini, L. Chesler

**Affiliations:** 1 Paediatric Tumour Biology, Division of Clinical Studies, The Institute of Cancer Research, London, UK; 2 Children and Young People’s Unit, Royal Marsden NHS Foundation Trust, London, UK; 3 Division of Radiotherapy and Imaging, The Institute of Cancer Research, London, UK

**Keywords:** Neuroblastoma, Telomere, Telomerase, MYCN, TERT, Alternative lengthening of telomeres

## Abstract

The majority of high-risk neuroblastomas can be divided into three distinct molecular subgroups defined by the presence of *MYCN* amplification, upstream *TERT* rearrangements or alternative lengthening of telomeres (ALT). The common defining feature of all three subgroups is altered telomere maintenance; *MYCN* amplification and upstream *TERT* rearrangements drive high levels of telomerase expression whereas ALT is a telomerase independent telomere maintenance mechanism. As all three telomere maintenance mechanisms are independently associated with poor outcomes, the development of strategies to selectively target either telomerase expressing or ALT cells holds great promise as a therapeutic approach that is applicable to the majority of children with aggressive disease.

Here we summarise the biology of telomere maintenance and the molecular drivers of aggressive neuroblastoma before describing the most promising therapeutic strategies to target both telomerase expressing and ALT cancers. For telomerase-expressing neuroblastoma the most promising targeted agent to date is 6-thio-2′-deoxyguanosine, however clinical development of this agent is required. In osteosarcoma cell lines with ALT, selective sensitivity to ATR inhibition has been reported. However, we present data showing that in fact ALT neuroblastoma cells are more resistant to the clinical ATR inhibitor AZD6738 compared to other neuroblastoma subtypes. More recently a number of additional candidate compounds have been shown to show selectivity for ALT cancers, such as Tetra-Pt (bpy), a compound targeting the telomeric G-quadruplex and pifithrin-α, a putative p53 inhibitor. Further pre-clinical evaluation of these compounds in neuroblastoma models is warranted.

In summary, telomere maintenance targeting strategies offer a significant opportunity to develop effective new therapies, applicable to a large proportion of children with high-risk neuroblastoma. In parallel to clinical development, more pre-clinical research specifically for neuroblastoma is urgently needed, if we are to improve survival for this common poor outcome tumour of childhood.

## Background

Neuroblastoma is a common childhood malignancy arising from the sympathetic nervous system. It most commonly arises from the adrenal gland and presents with an abdominal mass, but has a heterogeneous clinical phenotype. Children aged less than 18 months often present with widespread metastases to the skin, liver and bone marrow (stage MS disease) [1], however this can spontaneously resolve without treatment. Conversely, distant metastatic spread in children aged greater than 18 months (stage M disease) is associated with an aggressive clinical phenotype and poor survival [1, 2].

Children aged > 18 months with stage M disease and/or amplification of the *MYCN* oncogene are classified as having clinical high-risk disease. High-risk neuroblastoma remains a major therapeutic challenge with survival rates of < 50% despite intensification of therapy [2, 3]. However, until recently, in the absence of *MYCN* amplification, the molecular drivers of aggressive disease were unknown.

In 2015 it was reported that aggressive neuroblastoma can be divided into 3 almost mutually exclusive subgroups with either *MYCN* amplification, rearrangements upstream to the telomerase reverse transcriptase (*TERT*) gene or alternative lengthening of telomeres (ALT) [4, 5]. Each subgroup is associated with the activation of a telomere maintenance mechanism (TMM) and poor outcomes. Conversely, it is thought that the absence of a TMM is associated with spontaneous regression and excellent survival [6].

Here we summarise TMMs in cancer, specifically focusing on the molecular alterations driving telomere maintenance in neuroblastoma, before describing potential novel therapeutic strategies to directly target TMMs for children with neuroblastoma.

### Biology of telomere maintenance

Telomeres are regions of repetitive nucleotide sequences (TTAGGG) located at the ends of chromosomes that protect chromosomes from DNA damage, unnecessary DNA repair and fusion with other chromosomes. In normal dividing cells, with each cell replication telomeres gradually shorten, until a critical level is reached, the Hayflick limit, after which cells undergo senescence [7]. This gradual shortening of telomeres associated with cellular aging is believed to be a protective mechanism against uncontrolled growth, preventing cancer development in humans and other mammals [8]. In keeping with this, the activation of a TMM to prevent the shortening of telomeres is necessary for the continued sustained proliferation of cancer cells and hence a hallmark of cancer [9].

Telomerase, a functional ribonucleoprotein enzyme complex, maintains telomere length by adding telomeric DNA repeats at the 3′ ends of linear chromosomes. It is a reverse transcriptase that consists of a catalytic protein subunit TERT, and an essential RNA component known as human telomerase RNA (hTR), encoded by *hTERC*. hTR acts as a template for the synthesis of telomere DNA and is involved in the catalysis, localisation, and assembly of the telomerase holoenzyme [10, 11]. Telomerase is widely expressed in human embryos between 16 and 20 weeks gestation, however by the early neonatal period telomerase activity can no longer be detected in most somatic tissues [12]. In contrast, the majority of cancers overexpress telomerase: in a systematic analysis of 31 tumour types, over-expression of TERT was identified in 73% of all cancers [13]. This was most commonly associated with genetic alterations in either the *TERT* gene/promoter or *TERT* promoter methylation.

ALT is defined as maintenance of telomeres in the absence of telomerase activity [14]. It can be detected in 10–15% cancers overall but is particularly prevalent in tumours of mesenchymal origin [14, 15]. There is a strong association between ALT and loss of function (LoF) genetic alterations in *ATRX* (Alpha Thalassemia mental Retardation-X linked) in multiple malignancies, including neuroblastoma [13, 16–18].

**Fig. 1:**
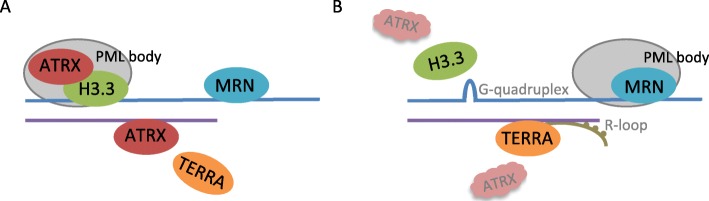
Mechanisms underlying the relationship between ATRX loss of function and ALT. Diagram of (**a**) a normal and (**b**) an ALT telomere. In normal cells ATRX and H3.3 co-localise with telomeric DNA, within PML bodies [23]. Following *ATRX* LoF, MRN complexes co-localise with PML bodies and a failure of telomeric H3.3 deposition results in G-quadruplex formation, facilitating non-canonical homologous recombination mechansims. Additionally, in the absence of functional ATRX, TERRA binds to telomeric DNA, facilitaing the formation of DNA-RNA hybrids known as R-loops which also promote homologous recombination repair

A number of different non-canonical homologous recombination (HR) based mechanisms have been proposed to play a role in ALT telomere maintenance [19–22]. Furthermore, a number of studies have focused on the underlying basis for the relationship between ATRX LoF and the development of the non-canonical HR mechanisms implicated in ALT (summarised in Fig. 1). Firstly an established role of ATRX is the maintenance of genomic stability via the deposition of H3.3 into telomeric regions [24, 25]. In the absence of ATRX, G4 quadruplex structures may occur in guanine rich regions of DNA such as telomeres, resulting in stalling of replication forks, which provides a substrate for HR [26, 27]. Secondly, in the absence of ATRX, the MRN (Mre11-RAD50-Nbs1) complex is redistributed to ALT associated PML body sites where it is thought to also facilitate HR mechanisms [26]. Finally, it has been shown that the long non-coding RNA TElomeric Repeat-containing RNA (TERRA) is functionally antagonistic with ATRX [28], and therefore in the absence of ATRX, TERRA can form DNA-RNA hybrids known as R loops, that promote homology directed repair of telomeres [29]. Further confirming the role of ATRX as an ALT repressor, *ATRX* knockdown has been shown to induce ALT activity in cells of mesenchymal origin [30]. However, *ATRX* depletion does not promote ALT activity in all cell types [31, 32] suggesting that ATRX LoF alone is not sufficient to induce ALT and that additional, as yet unidentified mechanisms are also needed. Reinforcing the notion that ALT arises as a result a combination of ATRX loss and other factors, it has recently been shown that during the immortalisation process, ATRX loss results in a progressive chromatin de-compaction and a gradual induction of telomere replication dysfunction which triggers an adaptive response eventually resulting in ALT activation [33]. Furthermore the authors show that the telomere dysfunction induced by ATRX loss cannot be overcome by endogenous telomerase activity.

Genetic alterations in the histone chaperone and ATRX binding partner *DAXX* (death domain associated protein) have also been shown to result in ALT due to a failure to localise ATRX to PML bodies [34]. In addition genetic alterations in tumour protein 53 (*TP53*) and retinoblastoma 1 (*RB1*) have also been associated with ALT [13] however the mechanistic basis for these associations is currently unknown.

### Biomarkers of TMM

In large-scale studies telomerase expression data is often used as a surrogate biomarker for telomerase activity [13], however quantification of telomerase activity by standardised assays based on the telomere repeat amplification (TRAP) protocol is also feasible in clinical trial settings both as a predictive biomarker in tumour tissue and as pharmacodynamic biomarker in peripheral blood mononuclear cells [35].

The identification of robust biomarkers of ALT has proved more challenging. The gold standard assay for ALT is to confirm the maintenance of telomeres, in the absence of telomerase activity through successive population doublings. However, this is only practical in cell lines, non quantitative, and requires long-term cell culture [14]. Although telomere length and telomere heterogeneity are often used as biomarkers of ALT the lack of specificity of these assays for ALT is becoming increasingly apparent [14, 36, 37].

PML bodies are ubiquitous throughout the genome and responsible for diverse functions including DNA repair. In ALT cancer cells PML bodies specifically co-localise to telomeric DNA, and are thought to facilitate HR [14, 26]. ALT associated PML bodies are now a well-established biomarker of ALT activity and have the advantage that they can be visualised in formalin fixed paraffin embedded (FFPE) material [14, 38].

ALT cells are also characterised by the presence of c-circles: single stranded, telomeric circular DNA strands which are thought to provide a template for ALT telomere synthesis [14]. The c-circle assay is a rolling circle PCR amplification assay, based on the self-priming nature of c-circles. The c-circle assay requires fresh-frozen tissue but is advantageous as c-circles are quantifiable, specific for ALT and c-circle levels can be used to evaluate response to ALT targeted agents [39, 40].

### Telomeres and telomere lengthening in neuroblastoma

Amplification of the *MYCN* oncogene is found in almost 40% of clinical high-risk neuroblastomas [4, 41], and is associated with up-regulation of TERT expression and telomere dysfunction [4, 42]. In an additional 23–31% of high-risk neuroblastomas, TERT is activated through chromosomal rearrangements involving 5p15.33, proximal to the *TERT* gene, which induces transcriptional up-regulation of *TERT* by juxtaposing the *TERT* coding sequence with strong enhancer elements [4, 5]. The third distinct sub-group, accounting for 24% of high-risk neuroblastoma cases are those with ALT [36]. Approximately half of ALT neuroblastomas are associated with somatic alterations in *ATRX* [4, 5, 36]. In neuroblastoma, genetic alterations in *ATRX* are associated with a distinct clinical phenotype including older age at diagnosis and a chronic progressive disease course [43].

**Fig. 2:**
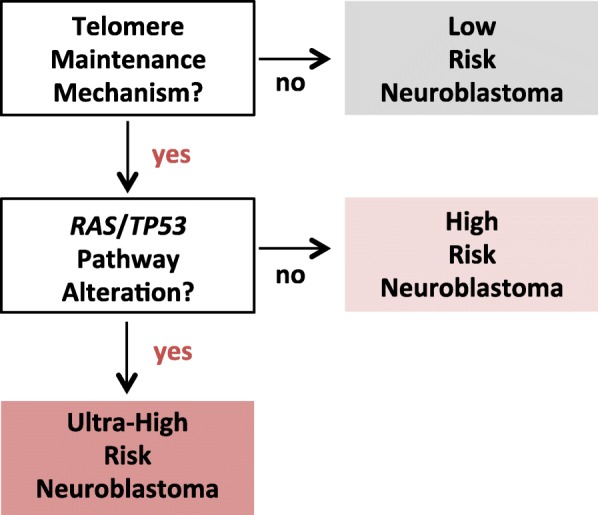
Molecular risk classification of neuroblastoma [6]. The presence of a telomere maintenance mechansim is defined as either *MYCN* amplification, a *TERT* rearrangement, telomerase upregulation or ALT. In neuroblastoma with a TMM the presence of a concurrent mutation in 1 of 17 defined *RAS* or *TP53* pathway genes defines a group of patients with particularly poor outcomes. The most common *RAS/TP53* pathway alterations found in neuroblastoma are activating ALK mutations

Neuroblastoma harbouring a TMM is associated with a poor prognosis regardless of clinical stage [4, 5, 44–46]. More recently it has been shown that in the presence of a TMM a concurrent mutation in a *TP53* or *RAS* pathway associated gene is associated with an even worse prognosis [6]. Conversely, neuroblastoma with *TP53* or *RAS* pathway mutations (including canonical ALK mutations) in the absence of a concurrent TMM are not associated with worse survival and can spontaneously regress [6]. This molecular risk stratification of neuroblastoma, as described by Ackermann et al. [6] is summarised in Fig. 2.

Although the somatic alterations driving TMM’s in the majority of neuroblastoma are well defined, high telomerase expression can occur in the absence of either a *TERT* translocation or *MYCN* amplification [6]. Also in the absence of an *ATRX* alteration the underlying drivers of ALT neuroblastoma are currently unknown, and somatic alterations in other genes, known to be associated with ALT in other malignancies are rarely found in neuroblastoma [47]. Numerous additional mechansims for telomere maintenance in neuroblastoma have been suggested. A study has identified interstitial telomeric sequences at sites of unbalanced translocations in neuroblastoma cell lines and postulated that these may contribute to a defective telomere maintenance pathway [48]. Another possibility is that genetic predisposition may contribute to the development of TMMs. In keeping with this, six common single nucleotide polymorphisms known to be associated with telomere lengthening have been found to be associated with an increased risk of neuroblastoma [49]. In keeping with the hypothesis that additional underlying predisposing factors may contribute to TMMs, a substantial increase in telomeric DNA damage and active telomere trimming has been described consistently across all high-risk neuroblastomas, regardless of telomerase or ALT status [50].

In summary, data is accumulating to support the hypothesis that TMMs are the common defining molecular feature of aggressive disease in the majority of children with clinical high-risk neuroblastoma. Therefore, both telomerase and ALT represent attractive targets for the development of novel therapeutic strategies with the potential to benefit a significant proportion of high-risk neuroblastoma patients. Here, we review the current pre-clinical and clinical research focused on targeting telomere maintenance with a specific focus on the potential relevance to patients with neuroblastoma.

### Drugs targeting telomerase activity

***Imetelstat (GRN163L)*** is a competitive telomerase inhibitor with a complementary structure to the template region of the RNA component of telomerase that binds to and blocks the active site of the enzyme [51]. Confirmation of target inhibition and pre-clinical efficacy of imetelstat has been demonstrated in multiple cancer subtypes [52–54].

A phase I trial of imetelstat in 20 children with refractory or recurrent solid tumours demonstrated telomerase inhibition, which was sustained through to day 8 of cycle 1 of therapy [55]. Two of 16 patients had a partial response, however no responses were seen in any of the 6 patients with neuroblastoma enrolled on the trial. The main dose limiting toxicities were neutropenia, thrombocytopenia, and lymphopenia. A Phase II study on imetelstat in children with recurrent or refractory central nervous system malignancies was also associated with significant haematological toxicity and was discontinued after two children died of intra-tumoral haemorrhage [35], thus paediatric development for this compound has subsequently been discontinued.

***BIBR1532*** is a potent synthetic, non-nucleoside telomerase inhibitor [56]. However like imetelstat there are significant concerns regarding toxicity [57], and this agent has not yet been evaluated in clinical trials.

***Sodium metaarsenite (KML001)*** binds to telomeric sequences, displacing hTERT from the nucleus into the cytoplasm [58] and has been shown to be cytotoxic in neuroblastoma cells in vitro [59]. In a phase I trial in adults with advanced solid tumours, objective responses to KML001 and cisplatin were seen in four out of 18 patients, however this trial was also discontinued due to toxicity [60].

***Telomestatin*** stabilises G-quadruplexes, which in turn, inhibits telomerase activity [61]. This compound has been shown to induce apoptosis in-vitro in telomerase expressing neuroblastoma cell lines [62] but is not yet in clinical development.

***6-thio-2′-deoxyguanosine (6-thio-dG)*** is a nucleoside analogue, which in telomerase active cells is recognised by telomerase and incorporated into telomeres resulting in telomere dysfunction [63]. Pre-clinical efficacy has been demonstrated in melanoma, non-small cell lung cancer and paediatric brain tumour models [64–66]. Although 6-thio-dG has not yet been evaluated in clinical trials, due to the novel mechanism of action of the compound it is thought to be less toxic than traditional telomerase inhibitors [67]. 6-thio-dG has been shown to be effective in-vivo in neuroblastoma models with *TERT* activation. However, the response in *MYCN* amplified xenografts was mixed, likely reflective of the additional oncogenic pathways activated by *MYCN* [47]. This is a priority compound for further development for neuroblastoma (Table 1).

**Table 1:** Compounds shown pre-clinically to target telomere maintenance mechanisms, to be prioritised for evaluation in neuroblastoma

Drug	Target	Pre-clinical Data	Clinical Trials
**6-thio-dG**	Telomerase	Pre-clinical efficacy in *TERT* activated neuroblastoma models	No
**Tetra-Pt (bpy)**	ALT	In-vitro and in-vivo activity in U2OS ALT osteosarcoma model	No
**CX-5461**	ALT	In-vitro *activity in U2OS and SAOS2 ALT osteosarcoma cell lines*	On-going adult phase I clinical trial (NCT0271997)
**Pifithrin-α**	ALT	In-vitro and in-vivo activity in U2OS ALT osteosarcoma model	No
**Trabectedin**	ALT	In-vitro activity in a panel of ALT cell lines (sarcoma, breast cancer and melanoma)	-FDA approval for certain soft tissue sarcomas [68] -Paediatric sarcoma phase II data [69] -On-going paediatric clinical trials (NCT04067115)

***XAV939*** is an inhibitor of tankyrase, a positive regulator or telomerase. It has been shown to induce apoptosis in the telomerase expressing neuroblastoma cell line SH-SY5Y [70].

### Drugs targeting ALT

#### Ataxia telangiectasia mutated (ATM) inhibitor combination therapy

It has been reported by Koneru et al. that ALT neuroblastoma cell lines are more resistant to topoisomerase inhibitors and that activation of ATM at ALT telomeres is associated with chemo-resistance. The ATM inhibitor AZD0156 was found to be synergistic with temozolomide and irinotecan therapy in both in vitro and in-vivo models of ALT neuroblastoma [71]. AZD0156 is currently in phase I trials in adults [72].

#### Ataxia telangiectasia and Rad3 related (ATR) inhibitor

**Fig. 3:**
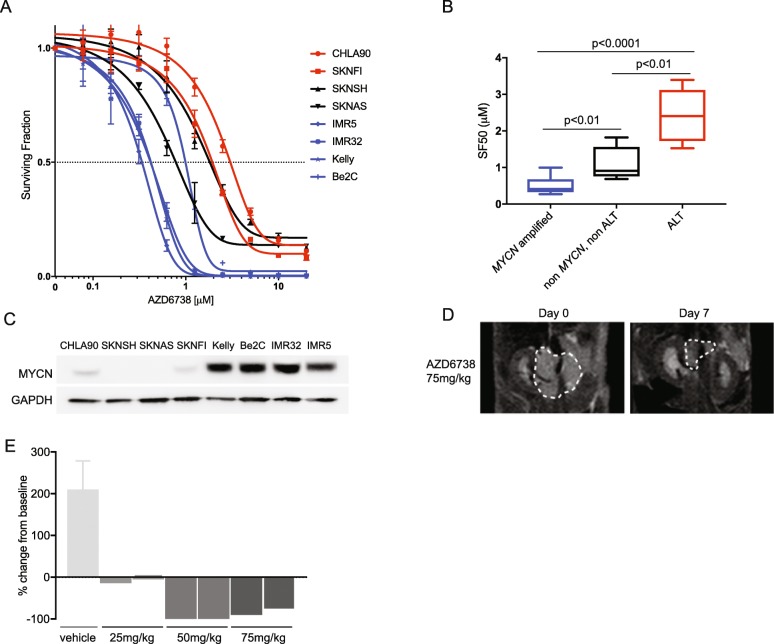
(**a**) Representative dose response curve for AZD6738 in a panel of neuroblastoma cell lines (**b**) Results of 3 independent SF50 experiments in the panel of neuroblastoma cell lines. Cell lines are grouped and colour coded according to MYCN and ALT [36, 75] status. *For SF*_*50*_*experiments, cells were seeded into 96-well plates and the following day compound was added to wells in triplicate, across a concentration gradient including DMSO-only controls. After 5 days cell viability was assessed by Cell Titer Glo****®****assay. The SF*_*50*_*was calculated as the drug concentration that inhibits viability/cell growth by 50% compared with controls, according to non-linear regression analysis, using Graphpad Prism. Statistical comparison of results is by unpaired t-test* (**c**) MYCN expression by western blot in the panel of cell lines *(N-MyC antibody: santa cruz sc-53,993, GAPDH antibody: cell signaling #2118)* (**d**) Representative images of a Th-*MYCN* GEMM tumour prior to, and after 7 days treatment with 75 mg/kg AZD6738. (**e**) Waterfall plot documenting the relative changes in tumour volume following 7-day treatment with AZD6738 at three different dose levels. *Preliminary studies of AZD6738 were performed in the Th-MYCN model of MYCN amplified neuroblastoma* [76]*. AZD6738 was supplied by AstraZeneca under a Material Transfer Agreement. It was diluted in 10% DMSO, 40% propylene glycol and 50% water as per manufacturers instructions. In a dose finding study three different doses (25 mg/kg, 50 mg/kg and 75 mg/kg) were trialled in 2 mice each for 7 days by oral gavage. Comparison of tumour response was made between animals receiving vehicle only and AZD6738. For response assessment, magnetic resonance imaging (MRI) of tumours was performed at day zero and after 7 days of administration of the compound*

In 2015 Flynn et al. reported that the presence of ALT renders cells hypersensitive to ATR inhibition in osteosarcoma models [73]. We have evaluated the clinical ATR inhibitor AZD6738 [74], in a panel of neuroblastoma cell lines and found that in contrast, ALT cell lines are relatively resistant to ATR inhibition in comparison to other neuroblastoma cell lines (Fig. 3a-b). This is in keeping with a subsequent publication which directly refuted the findings of Flynn et al. and concluded that differences in ATR inhibitor sensitivity were not related to ALT [77]. Taken together this data does not currently support a role of ATR inhibitors as an ALT specific therapy.

Conversely, we found that *MYCN* amplified neuroblastoma cell lines show a significantly greater in vitro sensitivity to single agent AZD6738 than non-*MYCN* amplified neuroblastoma cell lines (Fig. 3a-c), and a preliminary dose finding study demonstrated sensitivity to single agent AZD6738 in the Th-*MYCN* transgenic mouse model of *MYCN* driven neuroblastoma [76] (Fig. 3d-e). This is in keeping with other cMYC driven tumours where oncogene driven replicative stress results in a reliance on ATR signalling [78, 79]. Also in keeping with this, the SKNAS neuroblastoma cell line is relatively sensitive to AZD6738 (Fig. 3a) and known to over-express cMYC [80]. In *MYCN* amplified neuroblastoma models, ATR has also been proposed to play a role in resolution of transcription/replication conflicts [81].

#### Pre-clinical research in other ALT cancers

Recent pre-clinical data focused on the therapeutic targeting of ALT in other cancer subtypes may give important insight into potential therapeutic vulnerabilities that can be exploited for ALT neuroblastoma (Table 1): ***Tetra-Pt (bpy)*** is a cisplatin derivative that inhibits telomeric homologous recombination by targeting the telomeric G-quadruplex and has been shown to inhibit growth of ALT-cell xenograft tumours in mice [82]. Thus far this agent has not entered clinical trials nor has been evaluated in neuroblastoma models. ***CX5461*** is an RNA polymerase I inhibitor which has been shown to selectively kill *ATRX* mutant cells due to its effects on ribosomal RNA transcription. In-vitro sensitivity in ALT cancer cell lines has been demonstrated [83]. It has also recently been reported that some ALT cancer cells rely on p53 and AKT activity to suppress apoptosis. Furthermore the authors go on to show that the p53 inhibitor ***pifithrin-α*** suppresses tumour growth in an ALT xenograft model [84] although others have shown that pifithrin-α is not a specific inhibitor of p53 [85], calling into question the underlying mechanism of the demonstrated pifithrin-α response. This strategy may however represent a relevant novel therapeutic opportunity for ALT neuroblastoma, particularly in view of the fact that the majority of neuroblastoma is *TP53* wild type [86]. Finally, preliminary data has shown that ALT cancer cell lines are in general more sensitive to ***trabectedin,*** although the mechanism for this is not clear [87].

### Tumour heterogeneity, evolution and TMM targeted therapeutics

In some other paediatric malignancies, ALT (identified by the presence of ALT associated PML bodies) and telomerase activation have been shown to co-exist in the same tumour [88, 89]. In neuroblastoma, intra-tumoural diversity of telomere length in individual tumours has been identified using quantitative telomere fluorescence in-situ hybridisation (FISH) [90], however this is not a sufficiently sensitive or specific biomarker of ALT activity [14, 36]. In fact, one study has identified a subset of neuroblastomas with extremely long telomeres in the absence of either telomerase activity or ALT (detected by c-circle assay and ALT associated PML bodies) [36]. Further studies have shown that although *TERT* alterations and *MYCN* amplification do co-exist in a small proportion of cases, there is no overlap between the telomerase expressing and ALT positive neuroblastoma [47]. This is in keeping with a recent publication also showing that *ATRX* mutations and *MYCN* amplification are synthetically lethal in neuroblastoma [91]. Taken together, the evidence in neuroblastoma to date is that the presence of either ALT or telomerase activation is associated with differing distinct genetic drivers and occurs in mutually exclusive nature [4, 5].

Although data thus far indicates that subgroups of neuroblastoma are driven by either telomerase or ALT activation, it is highly likely that a selective pressure targeting one TMM will support the emergence of an alternative mechanism. In multiple other cancer subtypes it has been shown that long-term telomerase inhibition results in the emergence of features consistent with ALT [92–94]. Conversely, with the development of ALT targeted therapeutics it is probable that an up-regulation of telomerase will be seen. However, encouragingly, following *ATRX* LoF, it appears that ALT is a necessary adaption for cancer cell survival, and that reactivation of telomerase activity cannot overcome endogenous telomere dysfunction [33].

Finally although it has been shown that activation of a TMM is the key determinant of poor outcome in neuroblastoma, the key drivers of TMM’s in neuroblastoma; *MYCN* amplification and genetic alterations in *ATRX* are also associated with distinct patterns of wider transcriptional activation which drive malignant transformation [91, 95, 96]. Furthermore it is known that the co-occurrence of RAS/TP53 pathway alterations with a TMM is associated with a particularly dismal outcome [6] and accordingly, alterations in the RAS and TP53 pathways are enriched at the time of neuroblastoma relapse [97, 98]. Therefore, TMM targeting strategies will only be beneficial when given in combination with agents targeting these key pathways and the evaluation of combination therapies is urgently needed.

## Conclusion

Despite evidence that telomere maintenance is a key driver of aggressive biology in neuroblastoma, clinical translation of novel therapeutics specifically targeting telomere maintenance remains extremely challenging. The only compound to make it into paediatric clinical trials so far, imetelstat is excessively toxic and pre-clinical data on other compounds targeting telomerase activity is extremely limited. The most promising telomerase targeting candidate to date is 6-thio-dG, however this agent is yet to make it into clinical trials. It must also be noted that in an aggressive malignancy such as neuroblastoma, rapid development of resistance to single-agent targeting of telomerase activation is highly likely and that combination therapies will be needed to overcome this. Also, in the case of *MYCN* amplified tumours, transcriptional up-regulation of TERT is only one of many oncogenic programmes up-regulated in *MYCN* amplified neuroblastoma cells.

For ALT driven cancers, there is preliminary data supporting specific roles of a handful of targeted therapeutic approaches but a dearth of robust evidence of pre-clinical efficacy specifically for neuroblastoma. The combination of ATM inhibition with chemotherapy is currently the most promising option for ALT neuroblastoma, although regimens combining cytotoxic chemotherapy agents and inhibition of master upstream regulators of DNA damage repair such as ATM are likely to be prone to significant toxicities.

Despite these challenges, the development of effective new strategies for neuroblastoma by either selective targeting of telomerase or ALT offers great potential to treat the underlying drivers of aggressive disease biology, and is applicable to the greater proportion of neuroblastoma patients. Furthermore, the hypothesis that TMM targeting strategies may be particularly effective in neuroblastoma is supported by the fact that significantly fewer mutations are found in neuroblastoma in comparison to adult malignancies, which often arise due to an accumulation of oncogenic mutations over time [99].

In addition, as recent data identifies an ‘ultra-high risk’ group of neuroblastoma patients with both telomere maintenance and mutations in *RAS* and *TP53* pathway genes [6], rational combinations of telomere targeting agents with other targeted therapeutics must be sought. As pre-clinical data develops, rationally designed paediatric clinical trials will be required to personalise therapy to simultaneously target multiple drivers of aggressive biology in an individual patient.

As our understanding of the molecular drivers of fatal neuroblastoma has significantly expanded in recent years, pre-clinical research efforts must now focus on translating this knowledge into effective, less toxic new therapies for children with neuroblastoma.

## Data Availability

Data sharing is not applicable to this article as no datasets were generated or analysed during the current study.

## Abbreviations

TERT: Telomerase reverse transcriptase
ALT: Alternative lengthening of telomeres
TMM: Telomere maintenance mechanism
hTR: Human telomerase RNA
LoF: Loss of function
ATRX: Alpha Thalassemia mental Retardation-X linked
HR: Homologous recombination
MRN: Mre11-RAD50-Nbs1
PML: Pro-myelocytic Leukaemia
TERRA: RNA TElomeric Repeat-containing RNA
DAXX: Death domain associated protein
TP53: Tumour protein 53
RB1: Retinoblastoma 1
TRAP: Telomere repeat amplification protocol
FFPE: Formalin fixed paraffin embedded
ATM: Ataxia telangiectasia mutated
ATR: Ataxia Telangiectasia and Rad3 related
FISH: Fluorescence in-situ hybridisation
